# Current and recent clinical trials for perioperative systemic therapy for muscle invasive bladder cancer: a systematic review

**DOI:** 10.1186/1471-2407-14-966

**Published:** 2014-12-16

**Authors:** Vishal Vashistha, David I Quinn, Tanya B Dorff, Siamak Daneshmand

**Affiliations:** Department of Internal Medicine, Cleveland Clinic Foundation, Cleveland, OH USA; Division of Oncology, USC/Norris Comprehensive Cancer Center, USC Institute of Urology, Los Angeles, CA USA; Department of Urology, USC/Norris Comprehensive Cancer Center, USC Institute of Urology, 1441 Eastlake Abe, Suite 7416, Los Angeles, CA 90089 USA

**Keywords:** Bladder cancer, Radical cystectomy, Perioperative chemotherapy, Perioperative targeted therapy, mTOR inhibitor, Immune therapy, Gene therapy, Vaccine therapy

## Abstract

**Background:**

Although Muscle Invasive Bladder Cancer (MIBC) is increasing in incidence, treatment has largely remained limited to radical cystectomy with or without cisplatin-based neoadjuvant and/or adjuvant chemotherapy. We reviewed the current and recent clinical trials evaluating perioperative chemotherapy, targeted therapy, and novel therapeutic regimens for MIBC patients undergoing radical cystectomy.

**Methods:**

An overview of perioperative MIBC management was conducted initially using MEDLINE. The Clinical Trials Registry and MEDLINE were further searched specifically for perioperative MIBC chemotherapy, targeted therapy, and other novel therapeutic approaches. Trials involving non-perioperative management, operative management other than radical cystectomy, multiple tumors, or purely superficial or metastatic disease were excluded from selection. These criteria were not specifically fulfilled for mTOR inhibitor and immune therapy trials. Only phase III chemotherapy and phase II targeted therapy trials found in the Clinical Trials Registry were selected. MEDLINE searches of specific treatments were limited to January 2009 to January 2014 whereas the Clinical Trials Registry search had no timeline. Systematic MEDLINE searches had no phase restrictions. Trials known by the authors to fulfill search criteria but were not found via searches were also selected.

**Results:**

Twenty-five trials were selected from the Clinical Trials Registry including 7 phase III chemotherapy trials, 11 Phase II targeted therapy trials, 3 immune therapy trials, 1 mammalian target of rapamycin (mTOR) inhibitor trial, and 3 gene and vaccine therapy trials. Nine trials have been completed and 5 have been terminated early or withdrawn. Nine trials have data available when individually searched using MEDLINE and/or Google. Systematic searches of MEDLINE separately found 12 trials in the past 5 years. Two phase III chemotherapy trials were selected based on knowledge by the authors. No phase III trials of targeted therapy have been registered or published.

**Conclusions:**

New trials are currently being conducted that may revolutionize MIBC treatment preceding or following cystectomy. Head-to-head phase III trials of perioperative chemotherapy and further phase II and phase III trials of targeted therapy and other therapeutic approaches are necessary before the current cisplatin-based perioperative chemotherapy paradigm is altered.

## Background

Bladder cancer is the 4^th^ most common cancer in men and 11^th^ most common cancer in women in the United States [1]. In total, 74, 690 new cases are diagnosed each year with 15, 580 deaths annually [1]. Though rarer worldwide compared to other cancers, bladder cancer is also increasing in incidence globally as the use of tobacco products becomes more prevalent in developing nations [2]. In the United States, about 30% of these tumors invade past both the bladder submucosa and mucosa and are therefore defined as Muscle Invasive Bladder Cancer (MIBC) [1].

Though MIBC is prevalent both in the United States and internationally, treatment options for MIBC have remained essentially unchanged for the past 25 years. Traditionally, radical cystectomy has been the mainstay, but as evidence accumulates for the benefit of perioperative therapy, neoadjuvant or adjuvant cisplatin-based chemotherapy have become more valid options for MIBC patients [3–13]. Despite this success and that of other trials, only 15-20% of patients will receive neoadjuvant chemotherapy, though its prevalence has been increasing over time [14]. The number of medical oncologists recommending perioperative chemotherapy to their patients has increased as almost 80% of 92 oncologists recently surveyed have suggested perioperative chemotherapy to their MIBC patients, albeit those physicians were well-versed in bladder cancer treatment while many patients fail to have access to such medical oncologists for socieoeconomic reasons [15]. To continue the increasing trend of utilizing perioperative chemotherapy, clinical studies should address the overarching issues of predicting which patients will more likely benefit from chemotherapy, identifying particular chemotherapy regimens for specific patient subsets, and developing more efficacious non-cisplatin based regimens for patients who are cisplatin-ineligible. In addition, there is a desperate need for continued exploration of novel therapeutic treatment options to help modernize the perioperative management of MIBC.

As of now, perioperative MIBC clinical research is mainly focused on selecting a more efficacious cisplatin-based regimen using head-to-head trials with a limited number of studies addressing regimens for cisplatin-ineligible patients. Novel therapeutic approaches including targeted therapy, mammalian target of rapamycin (mTOR) inhibitors, immune therapy, and gene and vaccine therapy are now being evaluated in early phase trials with cautious optimism. Understanding the rationale and outcomes of these current and recent trials is imperative for clinicians and investigators to continue to encourage patient participation in these research efforts and to design future studies that enhance our ability to offer personalized treatment for MIBC patients. The following is a systematic review of the current and recent perioperative clinical trials conducted worldwide in the management of MIBC for patients undergoing radical cystectomy with an evaluation of specific areas that could benefit from future trials.

## Methods

### Data sources

Two separate databases were used to explore current and recent clinical trials for the perioperative management of MIBC. Initially, a Medical Literature Analysis and Retrieval System Online (MEDLINE) search through PubMed was completed for a general overview of the literature. This was followed by a search of all perioperative trials for MIBC using the Clinical Trials Registry online with the search phrase of “bladder cancer”. All trials selected for further review from the Clinical Trials Registry were independently searched using MEDLINE and Google for published results. The initial overview of MEDLINE abstracts and the Clinical Trials Registry search were used to develop individual search equations based on the different perioperative treatment classes for MIBC. Subsequently, MEDLINE was systematically searched for perioperative trials using these search equations. All search equations for MEDLINE and the Clinical Trials Registry are listed in Table 1. Lastly, any upcoming trials that are currently unregistered with the Clinical Trials Registry but were known by the authors to have been presented at any major urology and/or oncology conference were included. No new human subject data was studied requiring approval from an institution’s ethics board.

**Table 1:** Current and recent perioperative clinical trial systematic searches for MIBC*

Search category	Database	Search equation used	Trials selected/reviewed
Broad overview of all published abstracts	MEDLINE	(“bladder cancer” OR “urothelial cancer” OR “urothelial cell carcinoma” OR “transitional cell carcinoma”) AND (“bladder resection” OR “radical cystectomy”) AND (“perioperative chemotherapy” OR “adjuvant chemotherapy” OR “neoadjuvant chemotherapy” OR “targeted therapy” OR “biologic” OR “immunotherapy” OR “gene” OR “vaccine” )	744 abstracts reviewed^
All registered clinical trials with the NIH	Clinical trials registry	bladder cancer	25/695^#^
Trials of neoadjuvant chemotherapy	MEDLINE	((neoadjuvant) AND (chemotherapy OR cisplatin OR gemcitabine OR carboplatin OR methotrexate OR vinblastine OR doxorubicin OR adriamycin OR paclitaxel OR ifosfamide OR abraxane)) AND (bladder cancer) AND (radical cystectomy)	5/187
Trials of adjuvant chemotherapy	MEDLINE	((adjuvant) AND (chemotherapy OR cisplatin OR gemcitabine OR carboplatin OR methotrexate OR vinblastine OR doxorubicin OR adriamycin OR paclitaxel OR ifosfamide OR abraxane)) AND (bladder cancer) AND (radical cystectomy)	5/259
Trials of EGFR inhibitors	MEDLINE	(egfr inhibitor OR cetuximab OR erlotinib OR gefitinib OR genistein OR her-2 inhibitor OR lapatinib OR MGAH22 OR panitumumab OR trastuzumab) AND (bladder cancer OR radical cystectomy)	1/89
Trials of VEGF inhibitors	MEDLINE	(vegf inhibitor OR bevacizumab OR ramucirumab OR trebananib OR ziv aflibercept) AND (bladder cancer OR radical cystectomy)	0/64
Trials of other Tyrosine Kinase inhibitors	MEDLINE	(tyrosine kinase inhibitor OR dasatinib OR pazopanib OR sorafenib OR sunitinib) AND (bladder cancer OR radical cystectomy)	1/106
Trials of mTOR inhibitors	MEDLINE	(mTOR inhibitor OR everolimus OR sirolimus OR temsirolimus OR rapamycin) AND (bladder cancer OR radical cystectomy)	0/84
Trials of immune therapy	MEDLINE	(Immune therapy OR interferon-alpha) AND (bladder cancer OR radical cystectomy) AND (phase)	1/45
Trials of gene and vaccine therapy	MEDLINE	(vaccine OR gene therapy) AND (bladder cancer OR radical cystectomy) AND (phase)	1/87

NIH = National Institute of Health.

MEDLINE = Medical Literature Analysis and Retrieval System Online.

*MEDLINE clinical trial abstracts were only reviewed dating from January 2009 to January 2014. Trials from the Clinical Trials Registry Online and the MEDLINE broad overview of the literature had no date restrictions.

^No trials were individually selected from the MEDLINE broad overview of the literature. Treatment classes and specific drugs were taken from this MEDLINE search and from the trials search of the Clinical Trials Registry to develop the systematic search equations for each individual treatment class.

^#^This result includes phase III perioperative chemotherapy trials, phase II targeted therapy trials, and clinical trials of all phases for mTOR inhibitors, immune therapy, and gene and vaccine therapy.

### Study selection

Trials involving non-perioperative management, perioperative treatment for multiple tumors in addition to bladder cancer, operative management other than radical cystectomy, unconfirmed operative management, measured outcomes solely of serum or urine biomarker concentration, or purely metastatic or non-invasive disease were excluded from selection. The exclusion criteria of “unconfirmed operative management” was not strictly met for trials obtained from the Clinical Trials Registry involving rapamycin (mTOR) inhibition and immune therapy due to the current limited number of trials in those fields.

Clinical trials of all phases were reviewed but only current and recent phase III trials of perioperative chemotherapy and phase II trials of perioperative targeted therapy found in the Clinical Trials Registry were selected and further discussed. Trials of all phases from other perioperative treatment options were selected and evaluated. Published MEDLINE clinical trials selected from the systematic review of each treatment class were limited to data published between January 2009 and January 2014. No phase restrictions were placed on these MEDLINE systematic searches. Published MEDLINE trial data obtained from independently searching individual trials found on the Clinical Trials Registry was reported regardless of publication date.

## Results

**Figure 1:**
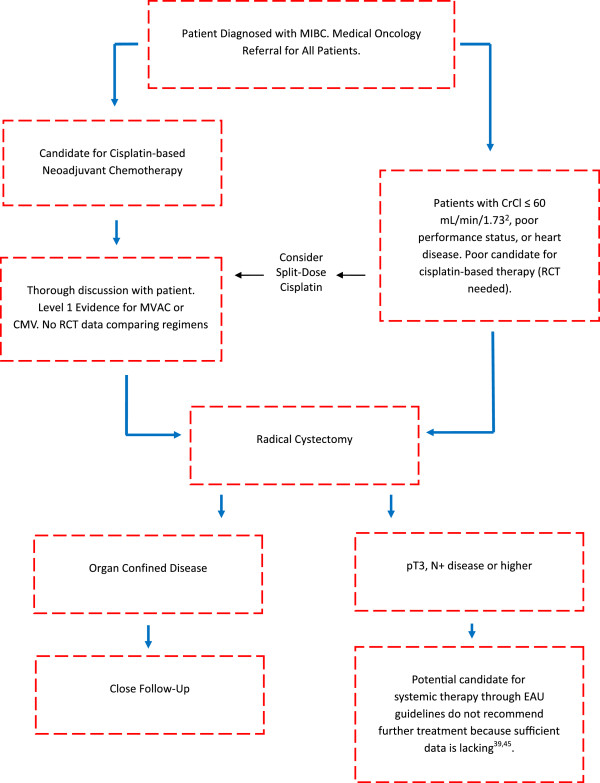
**Flow-chart of current management paradigm for patients with MIBC. RCT = Randomized Controlled Trial.**

The current treatment paradigm for MIBC management is conveyed in Figure 1. Outcomes from meta-analyses of previous phase III perioperative chemotherapy trials are reported in Table 2. The current and recent perioperative clinical trials for MIBC are identified below based on drug class. The classes include perioperative chemotherapy (Table 3), targeted therapy (Table 4), mTOR inhibition (Table 5), immune therapy (Table 5), gene therapy (Table 6), and vaccine therapy (Table 6).

**Table 2:** **Meta-analyses of perioperative chemotherapy phase III trials for MIBC**

Study	Treatment type	Year	Number of trials	Patients	Therapy	OS (HR)	DFS (HR)
Tjokrowidjaja et al. [16]	Neoadjuvant + Adjuvant	2013	21	3986	Only abstract available	0.86	0.77
						(0.79 – 0.93)	(0.71 – 0.84)
Tjokrowidjaja et al. [16]	Neoadjuvant	2013	12	3047	Only abstract available	0.89	0.80
						(0.81 – 0.98)	(0.73 – 0.88)
Advanced bladder cancer- Meta-analysis Corporation [17]	Neoadjuvant	2005	11	3005	Platinum-based	0.86	0.78
						(0.77 – 0.95)	(0.77 –0.95)
Winquist et al. [18]	Neoadjuvant	2004	11^^^	2605	Cisplatin-based	0.90	Insufficient data for analysis^+^
						(0.82 – 0.99)	
Tjokrowidjaja et al. [16]	Adjuvant	2013	9	939	Only abstract available	0.75	0.77
						(0.63 – 0.90)	(0.71 – 0.84)
Leow et al. [19] (Update of the meta-analysis below)	Adjuvant	2013	9^#^	945	Cisplatin-based	0.77	0.66
						(0.59 – 0.99)	(0.45 – 0.91)
Advanced bladder cancer meta-analysis corporation [20]	Adjuvant	2005	6*	491	Cisplatin-based	0.75	0.68
						(0.60 – 0.96)	(0.53 – 0.89)

OS: Overall Survival. DFS: Disease Free Survival. HR: Hazard Ratio.

^^^11 trials had HR data for OS and only 8 trials had HR data for DFS.

^+^Less than half the number of patients assessed were available for DFS results, and therefore, no statistical pooling analysis was undertaken.

^#^9 trials had HR data for OS and only 7 trials had HR data for DFS.

*6 trials had HR data for OS and only 5 trials had HR data for DFS.

**Table 3:** **Current and recent perioperative chemotherapy phase III trials for MIBC**

Institution	Regimen	Setting	Status/Results	Trial ID
University Hospital, Rouen	HD-MVAC vs. GC	Neoadjuvant or Adjuvant	Recruiting participants.	NCT01812369
Southwest Oncology Group	DD-MVAC vs. GC	Neoadjuvant	Recruiting participants [21].	
Japan Clinical Oncology Group	MVAC vs. Immediate RC	Neoadjuvant	Completed. 130 total subjects. OS increased by 35% in MVAC arm (64 subjects) but was not statistically significant. Improved pT0 rate with MVAC [22].	
FTRC	GC vs. Immediate RC	Adjuvant	Completed. 192 total subjects. No difference between adjuvant treatment (102) and control arms for 5 year OS. Failed to reach accrual goal, low study power [23].	NCT00054626
Cairo University	GC + radiotherapy vs. radiotherapy alone	Adjuvant	Completed. 146 total subjects. Improvement in 45-month DFS from 28% to 70% in chemoradiotheraphy (72) group but failed to be statistically significant [24].	NCT01734798
Eastern Cooperative Oncology Group	MVAC vs. PCa	Adjuvant	Completed. Results unreported.	NCT00003701
Sun Yat-Sen University	Intraarterial GC	Adjuvant	Recruiting Participants.	NCT01627197
Association of Urogenital Oncology	Adjuvant vs. Progression-Triggered Gemcitabine	Adjuvant	Completed. 114 total subjects. No statistically significant difference between adjuvant or progression-triggered Gemcitabine for DFS or OS. Failed to reach accrual goal [25].	NCT00146276
EORTC	Adjuvant vs. Progression Triggered MVAC + Gemcitabine	Adjuvant	Closed early due to poor accrual	NCT00028756

FTRC = Fondazione C.N.R./Regione Toscana “G. Monasterio”, Pisa, Italy. EORTC = European Organisation of Research and Treatment of Cancer. GC = Gemcitabine + Cisplatin. MVAC = Methotrexate + Vinblastine + Doxorubicin + Cisplatin. RC = Radical Cystectomy. PCa = Paclitaxel + Carboplatin. HD = High Dose. DD = Dose Dense. OS = Overall Survival. DFS = Disease Free Survival.

**Table 4:** **Current and recent perioperative targeted therapy phase II trials for MIBC**

Institution	Regimen	Receptor target	Setting	Results/Status	Trial ID
University of North Carolina	Erlotinib	EGFR	Neoadjuvant	Completed. Well tolerated. 35% (7/20) histologic downstaging preoperatively [26].	
University of North Carolina	Erlotinib	EGFR	Neoadjuvant + Adjuvant	Ongoing	NCT00380029
MDACC	Erlotinib	EGFR	Neoadjuvant	Ongoing	NCT00749892
Dendreon	DN24-02	HER2R	Adjuvant	Ongoing. Updated results: 75% of 226 patients had HER2 expression in primary tumor, 84% in lymph nodes. APC activation for all 30 patients who have received 3 infusions of drug [27].	NCT01353222
Medical University of South Carolina	Bevacizumab + GC and Bevacizumab + Paclitaxel^#^	VEGFR-A	Neoadjuvant + Adjuvant	Ongoing. Preliminary results: 42% (5/12) postoperative complication rate. 31% (4/13) downstaging preoperatively [28].	NCT00268450
MDACC	Bevacizumab + MVAC	VEGFR-A	Neoadjuvant	Ongoing	NCT00506155
FTRC	Sorafenib + GC	VEGFR + PDGFR*	Neoadjuvant	Unknown.	NCT01222676
US Oncology	Sunitinib + GC	VEGFR + PDGFR*	Neoadjuvant	Terminated early due to patient toxicity. Only 9 MIBC patients studied for neoadjuvant therapy [29].	
MSKCC	Sunitinib + GC	VEGFR + PDGFR*	Neoadjuvant	Completed early due to limited study accrual. pT0 rate was low with combination [30].	NCT00847015
CCCC	Sunitinib	VEGFR + PDGFR*	Neoadjuvant	Completed, Results Unreported	NCT00526656
Hoosier Oncology Group	Dasatinib	BCR/Abl^	Neoadjuvant	Completed. Well tolerated. Follow-up pathology study: SFK expression downregulated in 77% (14/18) of patients [31, 32].	NCT00706641
University of Michigan	Sunitinib	VEGFR + PDGFR*	Adjuvant	Terminated due to poor accrual	NCT01042795
Hoosier Oncology Group	Sunitinib + GC	VEGFR + PDGFR*	Neoadjuvant	Terminated due to patient toxicities	NCT00859339

MDACC = M.D. Anderson Cancer Center. FTRC = Fondazione C.N.R./Regione Toscana “G. Monasterio”, Pisa, Italy. MSKCC = Memorial Sloan-Kettering Cancer Center. CCCC = Case Comprehensive Cancer Center. GC = Gemcitabine and Cisplain. MVAC = Methotrexate, Vinblastine, Doxorubicin, Cisplatin. SFK = SRC Family Kinase. APC: Antigen Presenting Cell.

*Sorafenib and Sunitinib target multiple receptor tyrosine kinases (RTKs) in addition to the aforementioned receptors.

^Dasatinib targets multiple tyrosine kinases.

^#^This trial includes neoadjuvant bevacizumab with GC followed by surgery and adjuvant bevacizumab and paclitaxel.

**Table 5:** Current mTOR inhibitor and immune therapy trials for MIBC*

Institution	Regimen	Setting	Trial description	Status	Trial ID
UTHSCSA	Sirolimus	Neoadjuvant	Phase 0 trial for preoperative treatment. Tissue evaluated before and after treatment.	Recruiting	NCT01827618
Hoosier oncology group	Everolimus +/− Paclitaxel	Unspecified^	Phase II trial for Cisplatin ineligible patients. Not a perioperative specific study.	Recruiting	NCT01215136
University of Washington	Sirolimus + GC	Unspecified^	Phase I/II trial for disease of any stage. Patients likely will undergo cystectomy.	Ongoing	NCT01938573
MDACC	INF-alpha	Prior to Biopsy	Phase 1 trial with TURBT conducted following treatment. Radical cystectomy may follow.	Ongoing	NCT00082719

GC = Gemcitabine + Cisplatin. UTHSCSA = The University of Texas Health Science Center at San Antonio. MDACC = M.D. Anderson Cancer Center. TURBT = Transurethral Resection of Bladder Tumor.

*Table does not include trials for multiple solid tumors that may include bladder cancer as a subset.

^Unspecified regimens can be given in the neoadjuvant, adjuvant, locally recurrent, or metastatic setting.

**Table 6:** Current and recent perioperative gene and vaccine therapy trials for MIBC*

Institution	Therapy	Trial description	Status/Outcomes	Trial ID
Uppsala University	Gene	Phase 1/IIa Intravesical instillation of AdCD40L that may improve anti-tumor immune response. Cystectomy followed phase 1 trial.	Completed. 8 patients with limited complications. Successful gene transfer detected in biopsies [33].	NCT00891748
Celldex Therapeutics	Vaccine	Phase II neoadjuvant and adjuvant administration of CDX-1307 vaccine with chemotherapy for bladder tumors expressing B-HCG protein for patients undergoing radical cystectomy.	Terminated due to lack of enrollment.	NCT01094496
MSKCC and NCI	Vaccine	Phase 1 adjuvant administration of NY-ESO-1 peptide vaccine with BCG + sargramostim for tumors expressing NY-ESO-1 or LAGE-1 antigens. Patients previously underwent radical cystectomy.	Competed. Results unreported.	NCT00070070

*Table does not include trials for multiple solid tumors that may include bladder cancer as a subset.

MSKCC = Memorial Sloan-Kettering Cancer Center.

NCI = National Cancer Institute.

### Perioperative chemotherapy trials

The initial MEDLINE review of 744 abstracts identified a 2005 meta-analysis for neoadjuvant chemotherapy of phase III trials, an updated 2013 meta-analysis for adjuvant chemotherapy of phase III trials, and a systematic review of neoadjuvant and adjuvant chemotherapy trials prior to September 2012 [17, 19, 34]. A 2004 neoadjuvant meta-analysis by Winquist et al., the original 2005 adjuvant meta-analysis by the Advanced Bladder Cancer Meta-Analysis Corporation prior to the 2013 update, and a 2013 neoadjuvant and adjuvant meta-analysis abstract by Tjokrowidjaja et al. were also reviewed but were not results of the original MEDLINE search [16, 18, 20]. The results of the neoadjuvant and adjuvant meta-analyses are described in Table 2. Individual current and recent phase III neoadjuvant and adjuvant chemotherapy trials are detailed in Table 3.

The Clinical Trials Registry search found a total of 12 neoadjuvant chemotherapy trials. These included 1 phase III trial, 10 phase II trials, and 1 phase 0 trial. The 1 phase III trial is a randomized control trial of high dose methotrexate, viblastine, doxorubicin, and cisplatin MVAC (HD-MVAC) compared to gemcitabine and cisplatin (GC) at The University Hospital, Rouen. This study is also accepting MIBC patients for adjuvant consideration. An additional phase III trial investigated by The Southwest Oncology Group has recently been registered involving a comparison of dose dense MVAC (DD-MVAC) and GC, though this was not found in our search [21]. Neither Phase III trial has available results.

The systematic review of MEDLINE of neoadjuvant chemotherapy identified 5 eligible trials out of 187 results. Two of these publications referred to one single-arm study of Gemcitabine with Carboplatin that resulted in safe, tolerable uptake of this regimen with a comparable efficacy to cisplatin-based regimens [35, 36]. Two other trials were subset studies of previous large phase III trials and are not described in Table 3 [37, 38]. The review also identified a phase II neoadjuvant erlotnib study that is discussed further below as a targeted therapy [26]. Lastly, a recently published phase III neoadjuvant MVAC trial by The Japan Clinical Oncology Group, which was more recent than our search timeline criteria, reported an improved overall survival (OS) with the neoadjuvant arm but was not statistically significant and is included in Table 3 [22].

A total of 9 adjuvant chemotherapy trials were found in the Clinical Trials Registry. This included 6 adjuvant-only phase III trials, 1 aforementioned neoadjuvant and adjuvant trial from The University Hospital, Rouen, and 2 phase II trials. One of these 6 adjuvant-only phase III trials, a National Cancer Institute trial of adjuvant MVAC with gemcitabine compared to progression-triggered MVAC with gemcitabine, was withdrawn prior to enrollment. A Google and MEDLINE search for each of the remaining 5 trials found results for 3 of these studies. Cognetti et al. at Fondazione C.N.R./Regione Toscana evaluated adjuvant GC vs. cystectomy alone and found no difference in OS, but the study failed to achieve its accrual goal [23]. A phase III trial at The Cairo University by Zaghloul et al. of GC with radiotherapy did show an increase in disease free survival (DFS) but failed to reach statistical significance [24]. The Association of Urogenital Oncology’s phase III trial of adjuvant gemcitabine compared to disease progression-triggered gemcitabine for cisplatin-ineligible patients found no statistically significant outcomes [25].

The systematic review of MEDLINE of adjuvant chemotherapy identified 5 eligible studies out of 259 results. One study is the aforementioned phase III trial conducted by Cognetti et al. and is included in the 2013 updated meta-analysis by Leow et al. [23]. One phase II trial showed benefit using adjuvant GC or MVAC compared to observation alone following cystectomy [12]. Three selected trials were neoadjuvant studies including 1 small single-arm study evaluating the efficacy and tolerability of a split-dosed cisplatin regimen for patients with renal impairment that was not found with the neoadjuvant chemotherapy trials systematic review [37–39].

### Perioperative targeted therapy trials

A total of 11 phase II targeted therapy trials were identified using the Clinical Trials Registry as detailed in Table 4. No phase III trials met study eligibility criteria. Seven were neoadjuvant trials, 2 were adjuvant trials, and 2 were both neoadjuvant and adjuvant trials. Of these 11 trials, 1 adjuvant trial of sunitinib by The University of Michigan was terminated due to poor accrual and 1 neoadjuvant trial of sunitinib with GC by The Hoosier Oncology Group was terminated due to patient toxicities. Five trials included combinations with chemotherapy with 1 trial being the neoadjuvant sunitinib trial that was terminated. A Google and MEDLINE search of each clinical trial found results for 3 studies. Preliminary results of a neoadjuvant trial of Bevacizumab and GC followed by adjuvant Bevacizumab and Paclitaxel at The Medical University of South Carolina have shown a high surgical complication rate though further results are pending [28]. A neoadjuvant trial of sunitinib and GC at Memorial-Sloan Kettering Cancer Center failed to show a significant rate of pT0 disease at surgery [30]. A trial of neoadjuvant dastanib by The Hoosier Oncology Group was well tolerated and further showed a marked decrease in tumor phosphorylated SRC Family Kinase (SFK) expression levels but failed to show changes in cell proliferation [31, 32].

The independent MEDLINE search of epidermal growth factor receptor (EGFR) inhibitors found 1 eligible study out of 89 results. This was a published phase II study involving neoadjuvant erlotinib at the University of North Carolina conveying erlotinib to be well-tolerated with a substantial pre-surgical downstaging rate [26].

Our MEDLINE search of vascular epithelial growth factor (VEGF) inhibitors provided 0 eligible trials out of 64 results.

Our search of other possible tyrosine kinase inhibitors provided 1 eligible trial out of 106 results. This was a neoadjuvant sunitinib with GC study published by US Oncology that was terminated early due to patient toxicities [29].

### Perioperative mTOR inhibitor trials

A total of 3 trials were selected from the Clinical Trials Registry involving mTOR inhibitors. These trials included a phase 0 neoadjuvant sirolimus trial by The University of Texas Health Science Center at San Antonio, a phase II everolimus with paclitaxel and carboplatin (PCa) trial by The Hoosier Oncology Group, and a phase I/II sirolimus with GC trial by The University of Washington. The phase II trial by The Hoosier Oncology Group is not a perioperative specific study. The phase I/II trial by The University of Washington includes patients that are likely to undergo cystectomy but will be further evaluated for surgery based on clinical judgment. The University of Washington phase I/II trial also involves non-invasive and metastatic disease.

Our MEDLINE search found 0 eligible trials out of 84 abstracts.

### Perioperative immune therapy trials

One immune therapy trial was selected from the Clinical Trials Registry as described in Table 5. This was a phase I trial of interferon-alpha being administered prior to transurethral resection of bladder tumor for patients who are potential candidates for radical cystectomy at The M.D. Anderson Cancer Center. The trial was not a perioperative specific study.

The MEDLINE search found 1 eligible trial out of 45 abstracts. This study was also found in the gene and vaccine therapy systematic review and is further discussed below and included in Table 6.

### Perioperative gene and vaccine therapy trials

1. phase I/II gene therapy trial was found using the Clinical Trials Registry. This was a completed study of the AdCD40L vaccine by Uppsala University, which was also found in the perioperative immune therapy MEDLINE search. 1 phase I vaccine therapy trial and 1 phase II vaccine therapy trial were found on the Clinical Trials Registry. The phase 1 trial sponsored by Memorial Sloan Kettering Cancer Center and The National Cancer Institute involving the adjuvant administration of NY-ESO-1 following radical cystectomy has been completed but the results remain unreported. The phase II trial sponsored by Celldex Therapeutics involving the neoadjuvant administration of a vaccine for beta-Human Chorionic Gonadotropin (bHCG) expressing MIBC tumors was terminated due to a lack of enrollment. The gene therapy and vaccine therapy trials are detailed in Table 6.

Our MEDLINE search for both gene therapy and vaccine therapy found 1 eligible trial out of 87 results. This was the published results of the AdCD40L immunogene administered as intravesical therapy prior to cystectomy conveying tolerable uptake of the treatment [20].

## Discussion

Thus far, the perioperative medical management of MIBC has largely been limited to platinum-based chemotherapy regimens, particularly involving cisplatin as described in Figure 1. Our systematic review identifies the current clinical trials evaluating perioperative chemotherapy regimens, targeted therapy, and other novel treatment options. Below, we address the benefits and limitations of these trials as well as highlight the need for further particular studies.

### Neoadjuvant chemotherapy trials

Though neoadjuvant chemotherapy has been proven beneficial, further research is necessary towards selecting a particular regimen based on patient demographics and tumor specifics particularly for patients who are cisplatin-ineligible. The 2005 meta-analysis conducted by The Advanced Bladder Cancer Meta-Analysis Collaboration found a 14% risk reduction in mortality or a 5% increase in 5-year OS with the use of neoadjuvant chemotherapy prior to radical cystectomy [17]. Over 90% of patients received cisplatin-based regimens [17]. Clinicians often substitute regularly-dosed cisplatin for split-dosed cisplatin or carboplatin for patients with medical comorbidities, mainly renal impairment. On the contrary, both the European Association of Urology (EAU) and The Society of Urologic Oncology recommend proceeding directly to radical cystectomy if patients cannot tolerate cisplatin-based treatment [40, 41]. Winquist et al. similarly found a 10% risk reduction in a 2004 meta-analysis of 11 trials with a 13% risk reduction or a 6.5% increase in OS in the 8 trials for patients undergoing a combination chemotherapy regimen [18]. All trials were cisplatin-based [18]. Lastly, Tjokrowidjaja et al. found a 11% risk reduction in the most recent 2013 meta-analysis, though only the abstract data is available and details of the patient population and 12 trials used have yet to be published [16]. Interestingly, no direct comparisons between neoadjuvant chemotherapy regimens have been completed in a phase III trial.

Our review identified 2 new phase III trials including HD-MVAC compared to GC at The University Hospital, Rouen and DD-MVAC compared to GC by The Southwest Oncology Group and 1 recently published phase III trial of MVAC by The Japan Clinical Oncology. The University Hospital, Rouen trial is both a neoadjuvant and adjuvant study with a goal of 500 total patients. The Southwest Oncology Group study is referred to as the Co-expression Extrapolation (CoXEN) trial because tumor messenger RNA, DNA, and stem cell biomarkers will be evaluated prior to administering MVAC or GC [21]. Choi et al. of The Southwest Oncology Group has previously shown that MIBC with protein properties to that of p53-mutated breast cancers have increased chemoresistance to cisplatin [42]. Additionally, other genomic characteristics of MIBC may help clinicians select an appropriate chemotherapy regimen. Therefore, the CoXEN trial can offer information as to which patients may benefit from chemotherapy and whether MVAC or GC is more appropriate based on tumor genetics. Overall, both The University Hospital, Rouen and The Southwest Oncology Group trials will offer insight into the safety profiles and efficacy of the two regimens when directly compared in a prospective randomized fashion. Neither of these trials compares chemotherapy regimens to a control group of cystectomy without neoadjuvant treatment. Lastly, Kitamura et al. of The Japanese Clinical Oncology Group found a 35% increase in OS for patients undergoing neoadjuvant MVAC compared to cystectomy-alone but did not quite reach statistical significance (p = 0.07) [22]. This study was closed early due to poor accrual and had a total of 64 patients in the neoadjuvant arm and 66 patients in the radical cystectomy-only arm [22]. Though the results were not statistically significant, this recently published trial offers continued cautious support for a cisplatin-based regimen in the neoadjuvant setting.

Addressing the role of neoadjuvant chemotherapy for cisplatin-ineligible patients has been difficult. Only 1 phase III neoadjuvant trial from 1997 has been conducted for carboplatin-based treatment [43]. Our MEDLINE review of neoadjuvant chemotherapy identified a single arm prospective trial of gemcitabine and carboplatin leading to a 24.1% (28/116) pT0 stage at surgery and an 89.7% OS rate with a median follow-up of 41 months [35, 36]. 44% of these patients were ruled cisplatin-ineligible [35, 36]. The study was hampered by a single arm design at a single institution with a shortened follow-up timeframe [35, 36]. Moreover, phase II carboplatin-based regimen trials have showed either no change in efficacy or worse efficacy than cisplatin-based regimens with varying levels of toxicity [44, 45]. Furthermore, our adjuvant chemotherapy MEDLINE review identified a phase II neoadjuvant split-dosed cisplatin study for MIBC patients [39]. The treatment regimen was well-tolerated with 11 of 23 patients achieving complete response to neoadjuvant treatment while 0 out of the 10 patients with chronic kidney disease had further decline in renal function [39]. The study was limited as only 6 patients underwent radical cystectomy while others preferred organ-sparing or palliative treatment due to age or comorbidities, but nevertheless, the split-dosed cisplatin regimen offers a possible alternative to traditional cisplatin-based therapy for patients who have renal impairment [39]. To our knowledge, no phase III split-dosed cisplatin trials have been conducted, thus reaffirming The EAU and The Society of Urologic Oncology’s stance for preceding directly to cystectomy for cisplatin-ineligible patients [40, 41].

The role of future trials in neoadjuvant chemotherapy should continue to assess particular regimens based on individual patient demographics and tumor specifics. Randomized, multi-center phase III trials for split-dosed cisplatin and carboplatin are also needed for possible evidence-based alternatives for patients who are unable to tolerate a traditional cisplatin-based regimen. Though meta-analysis data has proven its efficacy, several questions still need to be investigated to improve the benefit of neoadjuvant chemotherapy on a personalized level.

### Adjuvant chemotherapy trials

Adjuvant chemotherapy for MIBC has recently been shown to be beneficial though a specific regimen based on patient characteristics has yet to be discerned. The updated 2013 meta-analysis by Leow et al. reported a 23% risk reduction in mortality with adjuvant chemotherapy [19]. All trials involved cisplatin-based regimens [19]. In total, only 945 patients were used in the meta-analysis compared to the 3005 patients and 3047 patients included in the most recent neoadjuvant chemotherapy meta-analyses [16, 17, 19]. Additionally, the results suggest that adjuvant chemotherapy may play a greater role in patients with lymph node positive (N+) disease [19]. The 2013 update by Leow et al. added 3 trials and updated 1 trial to the original 6 trials included in the 2005 meta-analysis by the Advanced Bladder Cancer Meta-Analysis Corporation [19, 20]. The original meta-analysis was limited by a low study power with only 491 patients included [20]. In a separate analysis, Tjokrowidjaja et al. reported a 25% risk reduction in mortality with 939 patients, which is consistent with the findings of Leow et al. [16, 20] The limited number of patients in all meta-analyses leads to difficulty towards recommending adjuvant therapy to all MIBC patients post-cystectomy. In fact, The EAU has yet to recommend adjuvant chemotherapy [40]. Recent commentary on the EAU guidelines by Sternberg et al. as well as the perioperative chemotherapy summary from The 2012 Society of Urologic Oncology Annual Meeting suggest that this is purely because the data on adjuvant chemotherapy is insufficient [45, 46].

Our review identified 6 phase III trials registered with the Clinical Trials Registry including 1 completed head-to-head trial, 3 trials involving GC, and 2 comparing a traditional adjuvant delivery with a disease progression-triggered schedule. The head-to-head trial is a comparison of MVAC with PCa completed by The Eastern Cooperative Oncology Group though the results have yet to be reported. To our knowledge, this is the first completed periopertive chemotherapy trial directly comparing two chemotherapy regimens. This trial can offer insight into taxane-based regimens compared to traditional cisplatin-based treatments. PCa has been studied in the past as a single-arm trial of 92 patients showing tolerable patient uptake with a 5-year OS of 28.9% [47]. A head-to-head comparison with a traditional regimen will shed light on the efficacy of a taxane-based regimen and may prove its value for cisplatin-ineligible patients.

Three of the adjuvant trials selected by our review involve the use of GC alone, GC with radiotherapy, or GC delivered via the intrarterial route. Cognetti et al. of Fondazione C.N.R./Regione Toscana evaluated adjuvant GC at tumor relapse compared to cystectomy alone and found a 5-year OS hazard ratio (HR) of 1.29 in the treatment arm but failed to reach statistical significance (p = 0.24) [23]. Only 194 patients were included and the study failed to reach its accrual goal forcing the study to be closed early [23]. This study is included in the 2013 meta-analysis by Leow et al., and to our knowledge, is the first completed adjuvant phase III trial of GC alone, which is a common regimen for neoadjuvant therapy [19]. Another phase III trial at The Cairo University by Zaghloul et al. compared adjuvant GC with radiotherapy with postoperative radiotherapy alone and showed an increase in 45-month DFS from 28 +/− 20% to 70 +/− 6% in the chemotherapy arm but failed to be statistically significant (p = 0.18) [24]. Chemoradiation has been proven effective previously as a bladder-sparing modality for MIBC in a pooled-analysis of phase II and phase III trials, but this publication by Zaghloul et al. is the first phase III trial evaluating chemoradiation in the post-cystectomy setting [48]. Though the findings were not statistically significant, the combination of adjuvant GC with radiotherapy improved DFS, which suggests that adjuvant chemoradiation should be studied more extensively in the future [24]. Lastly, a current phase III study by Sun Yat-Sun University is evaluating the use of 1–3 cycles of intrarterial GC for adjuvant use within 1–5 weeks following cystectomy. Previously, a retrospective trial comparing 25 patients receving intraarterial GC with 35 patients solely undergoing cystectomy has recently been published by Jiang et al. [49] The data from the retrospective study is promising as the HR for 1-year survival dropped to 0.18 (p = 0.04) in the adjuvant treatment arm with the most common adverse effect being transient myelosuppresion (40%) [49]. A phase III trial of such a delivery method will shed better insight into the intraarterial options for adjuvant chemotherapy.

The last two adjuvant chemotherapy trials identified by our review investigate the role of disease progression-triggered delivery of adjuvant chemotherapy compared to a traditional adjuvant delivery. The Association of Urogenital Oncology phase III trial of adjuvant gemcitabine compared to disease progression-triggered gemcitabine for cisplatin-ineligible patients showed an increase in DFS (HR 1.375), cancer specific survival (HR 1.166), and 3-year OS (HR 1.225), but failed to show statistical significance for any of these outcomes (p = 0.335, p = 0.622, p = 0.426, respectively) [25]. Of note, the trial closed early and included 114 patients for analysis rather than the planned 178 patients [25]. Nonetheless, the trial offered an alternative to cisplatin-based treatment with a novel delivery time. Lastly, the Eastern Organisation for Research and Treatment of Cancer phase III trial of adjuvant MVAC vs. disease progression-triggered MVAC was withdrawn prior to enrollment. Currently, evidence fails to suggest that a non-cisplatin based regimen or a progression-triggered treatment is beneficial in the adjuvant setting.

Neoadjuvant chemotherapy has thus far been more thoroughly studied than adjuvant chemotherapy as highlighted by the larger number of patients included in the neoadjuvant meta-analyses. Regardless, similar to the current need in neoadjuvant chemotherapy clinical research for MIBC, more head-to-head phase III adjuvant chemotherapy trials are necessary to understand the benefits and drawbacks of particular regimens with the hopes of offering a certain combination based on patient and tumor specifics. Additionally, further trials particularly studying N+ patients can offer insight into the benefit of adjuvant chemotherapy. One previous phase II trial conveyed that GC is a tolerable adjuvant regimen for N+ disease while a phase III trial included in the updated 2013 meta-analysis by Leow et al. concluded that a cisplatin-based adjuvant regimen can particularly benefit patients with > pT3 and/or N+ disease [19, 50, 51]. Focusing phase III trials on this particular group of patients may define a specific role for adjuvant chemotherapy. Finally, current shortcomings of adjuvant trials are likely due to poor accrual forcing studies to close early. Clinicians and investigators need to continue to encourage patients to participate in these trials to improve our understanding of adjuvant chemotherapy.

### Perioperative targeted therapy trials

Thus far, the role of targeted therapy in the perioperative management of MIBC is convoluted because of the limited data from clinical studies. No active phase III trials of perioperative targeted therapy were identified by our systematic review. Our review identified 11 currently active or recently completed phase II trials. Eight of these studies are purely neoadjuvant with 2 other trials evaluating a combination of neoadjuvant and adjuvant therapy. This discrepancy between the number of neoadjuvant and advjuvant targeted therapy trials may be attributed to neoadjuvant chemotherapy being considered beneficial for a much longer time and with larger phase III studies than adjuvant chemotherapy. Five trials include targeted therapy combination treatment with chemotherapy. For 4 of these trials, the chemotherapy selected for combination treatment include traditional cisplatin-based regimens, which is expected as cisplatin-based regimens dominate the options for perioperative chemotherapy for MIBC. Of note, the Dendreon adjuvant study of DN24-02, which is designed to stimulate an immune response against the human epidermal growth factor receptor 2 (HER2) is discussed below as an immune therapy.

Erlotinib may be considered the best candidate for a future phase III trial of targeted therapy. Unlike other targeted therapies, erlotinib is mainly being evaluated alone without combination chemotherapy. A phase II trial of neoadjuvant erlotinib with 20 clinical T2 patients at The University of North Carolina led to 7 (35%) patients being downstaged to < pT1 at cystectomy without significant toxicity [26]. Since this trial was not a combination treatment trial with chemotherapy, the results convey that erlotinib alone can possibly offer neoadjuvant intervention. Two active erlotinib trials are currently being investigated by The University of North Carolina and M.D. Anderson Cancer Center, which will further offer insight on the safety and efficacy of erlotinib alone for neoadjuvant MIBC.

Multiple phase II trials of bevacizumab with chemotherapy are assessing the role of the VEGF inhibitor in perioperative MIBC management. A phase II trial by The Medical University of South Carolina evaluating bevacizumab with GC in the neoadjuvant setting and bevacizumab with paclitaxel in the adjuvant setting reported a 31% downstaging rate with a 42% postoperative complication rate for 13 patients in 2011 [28]. All patients underwent neoadjuvant therapy but only 2 patients underwent adjuvant treatment, and therefore, the effect of paclitaxel with bevacizumab cannot yet be predicted [28]. Postoperative complications included enterovesical fistula, ileus, and pelvic abscess [28]. Further patient accrual for the study will help describe whether the downstaging rate will be beneficial enough to use bevacizumab despite its high surgical complication risk once more results are reported. In addition, bevacizumab is currently being evaluated with MVAC by M.D. Anderson Cancer Center, which will further offer outcomes of the added treatment. Further information is needed currently for bevacizumab before a phase III trial can be undertaken.

Five of our 12 reviewed phase II targeted therapy trials involve sunitinib, but 2 of these studies by The University of Michigan and The Hoosier Oncology Group have been withdrawn due to poor accrual or patient toxicities, respectively. A trial of sunitinib with GC by US Oncology was also terminated early due to a hematologic toxicity rate of 70% despite lower dosing of GC to offset early adverse effects [29]. Only 9 MIBC cases were evaluated prior to radical cystectomy, but 22% (2/9) patients did have complete pathologic response to treatment [18]. The same regimen was studied at Memorial Sloan-Kettering Cancer Center and was hampered by a low pT0 rate of 31% (4/13), which is a slightly decreased rate to that of GC or MVAC neoadjuvant therapy alone, thus suggesting a limited efficacy of the added sunitinib [4, 30, 52]. Case Comprehensive Cancer Center has recently completed a trial of neoadjuvant sunitinib alone, which will hopefully assess whether sunitinib can have a role monotherapy, but the results remain unreported. Neoadjuvant sunitinib has not been proven beneficial at this time either as a monotherapy or as a combination therapy and evidence does not currently warrant a phase III trial.

Our review also identified 2 trials involving dasatinib and sorafenib as possible perioperative targeted therapies. A phase II trial of dasatanib monotherapy by The Hoosier Oncology Group of 25 patients was tolerated by 68% with 4% undergoing hematologic toxicity [31]. The follow-up pathology results conveyed a decrease in phosphorylated SFK expression in 77% (14/18) of patients but failed to show a change in cell proliferation or apoptosis while not identifying a downstaging rate [32]. Phosphorylated SFK expression is a common and specific histologic manifestation of bladder cancer [32]. Further investigation is required to understand how dastanib’s effect on phosphorylated SFK expression can alter patient outcomes and if this actually has any clinical role towards treating MIBC. Lastly, our review found a sorafenib with GC study at Fondazione C.N.R./Regione Toscana, though the status of this study remains unreported.

The next step towards considering targeted therapy as a legitimate option for the perioperative management of MIBC would be a phase III trial. The most feasible option would be to await the results of the 2 current erlotinib clinical trials at The University of North Carolina and The M.D. Anderson Cancer Center. Based on the findings from these studies in addition to the published results from a previous University of North Carolina neoadjuvant erlotinib trial, erlotinib alone could be a candidate for a phase III trial [26]. Further phase II trials of neoadjuvant dasatinib addressing tumor staging following treatment could lead to a phase III trial. As of now, an appropriate regimen for a phase III trial of chemotherapy and targeted therapy would be difficult to elucidate based on the current data.

### Perioperative mTOR inhibitor trials

The mTOR protein serves as a regulator for cell growth and proliferation based on stimuli stemming from nutrient availability and energy processes within the cell [53]. The upregulation of this protein has been shown in solid tumors and hematologic malignancies, and therefore, inhibition of the mTOR protein can limit malignancy growth for patients refractory to other treatments [53]. Consequently, the role of mTOR inhibitors is now being evaluated in bladder cancer. Everolimus has been shown to affect bladder cancer lines in vitro and in mouse models [54]. Additionally everolimus has been combined with cisplatin to decrease cell proliferation in vitro [55]. Temsirolimus and Everolimus have been studied as phase II trials in metastatic urothelial cancer [56, 57]. However, no perioperative specific trials for MIBC have been completed.

Our review identified 1 phase 0 neoadjuvant trial of sirolimus at The University of Texas Health Science Center at San Antonio. This trial is of particular importance because it can offer evidence that mTOR inhibitors can be safely tolerated in patients undergoing a major cystectomy operation. Additionally, despite failing to fulfill criteria as being perioperative specific studies, we selected an everolimus with paclitaxel trial by The Hoosier Oncology Group and a sirolimus with GC trial by The University of Washington. These trials were selected because of the lack of current available mTOR inhibitor trials. The everolimus with paclitaxel phase II trial is a first-line therapy for invasive bladder cancer for patients who are not candidates for cisplatin-based treatment. The sirolimus with GC trial is a phase I/II study for patients who are likely to undergo cystectomy, thus it can be considered a neoadjuvant study, but surgical intervention is not definitive based on the Clinical Trials Registry information. The results from these studies may promote pure perioperative studies of mTOR inhibitors in the future.

Further perioperative phase I and phase II trials of mTOR inhibitors for MIBC are imperative to encourage their use. As of now, no real clinical data can define the role of mTOR inhibitors for MIBC patients undergoing cystectomy.

### Perioperative immune therapy trials

Immune therapy has yet to be thoroughly studied in bladder cancer. A large phase II trial has been previously conducted evaluating interferon-alpha 2B with The Bacillus Calmette–Guérin (BCG) vaccine in non-invasive bladder cancer [58]. Interferon-alpha-2B has further been studied independently for non-invasive bladder cancer as intravesical administration for patients who failed BCG therapy [27]. However, no trials have been completed examining immune therapy for MIBC.

Despite not fulfilling criteria for being a perioperative specific study, we selected a trial of interferon-alpha for advanced bladder cancer at M.D. Anderson Cancer Center. Evaluating the tolerability of interferon-alpha in advanced bladder cancer can help define the safety profile of the treatment. The trial was selected due to the scarcity of immune therapy trials for MIBC. In addition, an ongoing trial reported in Table 4 by Dendreon of DN24-02, an immune stimulant against HER2, includes the use of culturing peripheral blood mononuclear cells with recombinant antigen BA7072 following 3 2-week cycles of leukopharesis and infuses this product back into the patient’s bloodstream [59]. BA7072 is a fusion protein of HER500, a protein that has intracellular and extracellular elements of HER2, and granulocyte-macrophage colony-stimulating factor, which enhances activation of antigen presenting cells (APC’s). Measured responses of the trial include serum APC activation, particularly BA7072 and HER500 specific antibody proliferations [59]. Thus far, 226 patients have been enrolled in this trial with 75% of these patients having some level of HER2 expression in the primary tumor and 84% in lymph node samples. Thirty patients have undergone the full 3 infusion cycles with APC activation evident after each infusion but responses were greater at infusions 2 and 3 than at infusion 1 [59]. Further long-term results can describe the actual effect of DN24-02 on clinical outcomes. On an overarching note, the administration of DN24-02 can be considered a mixture of targeted therapy with immune therapy considerations. Given the heterogeneity of bladder cancer biology, and the clear need for less toxic, non-cisplatin based therapies and therapies for patients in whom neoadjuvant chemotherapy has failed, the fact that this phase II trial actually met its accrual goal should be celebrated as enthusiasm for rational targeted therapy by investigators for MIBC.

Moving forward, perioperative specific trials of interferon-alpha and a further phase III trial of DN24-02 are necessary to understand their efficacy and tolerability among patients undergoing cystectomy. The data for immune therapy is currently too limited to suggest its benefit in MIBC.

### Perioperative gene and vaccine therapy trials

Gene and vaccine therapy trials for MIBC are being evaluated for patient tolerability. A phase I/IIa trial of AdCD40L by Uppsala University used an intravesical administration of an adenovirus vector to upregulate the human CD40 ligand in MIBC patients scheduled for cystectomy [33]. Only 1 out of 8 patients experienced a serious adverse effect postoperatively, and the histologic samples showed increased immune activation and decreased cell proliferation with successful gene transfer [33]. Though only an early phase trial, the study conveyed that gene therapy can be well tolerated prior to cystectomy.

In addition to the AdCD40L trial, our review identified two other current studies in gene and vaccine therapy. Celldex Therapeutics terminated a phase II trial of neoadjuvant and adjuvant CDX-1307 with chemotherapy for MIBC patients with Newly Diagnosed Muscle-Invasive Bladder Cancer (The N-ABLE Trial). The study was terminated due to poor accrual. CDX-1307 is a monoclonal antibody targeting the mannose receptor and bHCG, a common couplet in epithelial tumors [60]. CDX-1307 had been studied previously in a phase I trial of multiple tumors showing tolerability of the vaccine and increased immune response [61]. The CDX-1307 phase II trial offered a novel combination of vaccine therapy and chemotherapy, and such a treatment regimen can be emulated in future studies. The trial’s failure to accrue its patient goal may be a reflection of a current lack of faith in vaccine or immune therapy for MIBC. Furthermore, Memorial Sloan-Kettering Cancer Center and The National Cancer Institute have completed a phase I vaccine trial of NY-ESO-1, BCG, and sargramostim in patients who recently underwent cystectomy for bladder cancer and express NY-ESO-1 and/or LAGE-1 antigens histologically. NY-ESO-1 and LAGE-1 are antigens expressed by testicular germ cells and a limited number of tumors; however, previous results indicate that almost 50% of high grade urothelial carcinomas express one or both of these antigens, and thus, a vaccine for either may prove beneficial in bladder cancer [62]. Previously, Sharma et al. have demonstrated tolerable uptake of the vaccine with BCG and granulocyte macrophage colony-stimulating factor for 6 patients that underwent cystectomy for MIBC [63]. NY-ESO-1 specific antibody responses were found in 5/6 patients with CD4 T-cell responses being found in 6/6 patients [63]. The new phase I study that has been completed had a project accrual of 24–28 patients and will shed more light on the tolerability of the vaccine for MIBC patients. The NY-ESO-1 vaccine has been used tested previously in 16 patients with non-small cell lung cancer, esophageal carcinoma, or prostate adenocarcinoma and was well-tolerated [64]. The clinical outcomes were mixed but it was noted that regulatory T cells in the blood may limit the antibodies produced by the vaccine and suppressing this immune response can possibly improve the vaccine’s effectiveness [63]. This is one of the shortcomings of the vaccine that requires further study. Nonetheless, identifying eligible patients for the NY-ESO-1 vaccine based on tumor specific antigens is a reflection of MIBC research moving towards personalized medicine.

The safety profile of gene and vaccine therapy has yet to be elucidated for any specific treatment. AdCD40L is a promising immune-stimulating agent, but a future phase II trial is necessary to further assess tolerability. CDX-1307 theoretically would have benefit in bHCG expressing bladder cancers; however, no bladder cancer specific trial has been completed thus far. Lastly, NY-ESO-1 and LAGE-1 offer targets for peptide vaccine therapy, but results from the current NY-ESO-1 vaccine trial and future clinical studies are required to assess the tolerability and efficacy of the treatment.

### Expanding areas for future clinical trials

The Cancer Genome Atlas (TCGA) Research Network is a widespread effort developed by the National Cancer Institute to collect specimens of several cancers with the hopes of analyzing the genetics and molecular biology of tumors to identify common mutations and targets for treatment [65]. Recently, TCGA published data on 131 MIBC specimens [66]. Mutations in 32 genes were found to be statistically significant, reinforcing current targets for clinical trials and suggesting new areas for therapeutic studies [66]. Using the data from the consortium to identify need for future study may prove beneficial towards constructing new trials.

### Limitations of our systematic review

All searches were limited to the Clinical Trials Registry, MEDLINE, and Google. Any abstract or current clinical trial unregistered or unreported by the aforementioned databases may have been overlooked unless specifically known of through recent conferences. However, our searches detailed an extensive review of multiple databases for perioperative MIBC trials.

Phase I and II trials of perioperative chemotherapy were not substantially discussed above because of the wide array of literature on the topic currently. Phase III perioperative chemotherapy subset studies of previous larger phase III trials were not substantially discussed because of overlap with trials included in past meta-analyses. Only non-subset phase III trials were evaluated in detail because of their possible changes in clinical management of perioperative MIBC in the near future. Similarly, phase I trials of targeted therapy were not closely addressed in comparison to phase II trials.

## Conclusion

Although the perioperative management of MIBC has been limited to platinum-based chemotherapy, several novel treatments are being evaluated for tolerability and efficacy. Phase III trials directly comparing chemotherapy regimens are now being studied, which will hopefully classify different regimens for specific patients. Targeted therapy with monoclonal antibodies is the closest non-chemotherapy treatment to being implemented, but no phase III trial of targeted therapy has been conducted. Further trials in all treatment modalities are required to address the large need for improved perioperative options in MIBC management.

## Abbreviations

MIBC: Muscle Invasive Bladder Cancer
MEDLINE: Medical Literature Analysis and Retrieval System Online
MVAC: Methotrexate, Vinblastine, Adriamycin (doxorubicin), and Cisplatin
GC: Gemcitabine and Cisplatin
PCa: Paclitaxel and Carboplatin
CMV: Cisplatin, Methotrexate, Vinblastine
HD: High Dose
DD: Dose Dense
SFK: Src Family Kinase
EGFR: Epidermal Growth Factor Receptor
VEGF: Vascular Epithelial Growth Factor
HER2R: Human Epidermal Growth Factor Receptor 2
BCG: Bacillus Calmette–Guérin
bHCG: beta-Human Chorionic Gonadotropin.
